# VscF in T3SS1 Helps to Translocate VPA0226 in *Vibrio parahaemolyticus*

**DOI:** 10.3389/fcimb.2021.652432

**Published:** 2021-04-01

**Authors:** Lele Lian, Jiao Xue, Wanjun Li, Jianluan Ren, Fang Tang, Yongjie Liu, Feng Xue, Jianjun Dai

**Affiliations:** MOE Joint International Research Laboratory of Animal Health and Food Safety, College of Veterinary Medicine, Nanjing Agricultural University, Nanjing, China

**Keywords:** *Vibrio parahaemolyticus*, type III secretion systems 1, needle protein, VscF, VPA0226

## Abstract

In *Vibrio parahaemolyticus*, type III secretion system 1 (T3SS1) is a major virulence factor that delivers effectors into the host eukaryotic cytoplasm; however, studies on its infection mechanism are currently limited. To determine the function of the *vscF* gene, we constructed the *vscF* deletion mutant Δ*vscF* and complementation strain CΔ*vscF*. Compared with those of wild-type POR-1 and CΔ*vscF*, the cytotoxic, adherent, and apoptotic abilities of Δ*vscF* in HeLa cells were significantly reduced (*P* < 0.01). Furthermore, in infected HeLa cells, the mutant strain reduced the translocation rates of VP1683 and VP1686 effectors compared to the wild-type and complementation strains. A BLAST search showed that *vscF* is homologous to the MixH needle protein of *Shigella flexneri*, indicating that the *vscF* gene encodes the needle protein of T3SS1 in *V. parahaemolyticus*. Additional translocation assays showed that VPA0226 translocated into the HeLa eukaryotic cytoplasm *via* T3SS1, secretion assays showed that VPA0226 can be secreted to supernatant by T3SS1, indicating that VPA0226 belongs to the unpublished class of T3SS1 effectors. In conclusion, our data indicate an essential role of vscF in *V. parahaemolyticus* T3SS1 and revealed that VPA0226 can be secreted into the host cell cytoplasm *via* T3SS1. This study provides insights into a previously unexplored aspect of T3SS1, which is expected to contribute to the understanding of its infection mechanism.

## Introduction

*Vibrio parahaemolyticus* is a gram-negative bacterium that often causes food poisoning in coastal areas and leads to gastroenteritis and septicemia (Daniels et al., 2000). With the improvement of living standards and increase in seafood consumption, it has become an important foodborne pathogen (Nair et al., 2007; Hu et al., 2020). The type III secretion system 1 (T3SS1) of *V. parahaemolyticus* is an essential virulence determinant and is encoded by the *vp1656-1702* gene cluster on chromosome 1 (Park et al., 2004b; Enninga and Rosenshine, 2009), which consists of a basal body and an extracellular needle complex.

In *Yersinia pestis*, current data have comprehensively demonstrated the secretion mechanism of T3SS. For example, some researchers (Payne and Straley, 1999; Hoiczyk and Blobel, 2001; Edqvist et al., 2003; Journet et al., 2003; Wood et al., 2008) studied and reported the assembly of T3SS of *Y. pestis*. However, to our knowledge, although Park et al. (2004b) have reported the clustering of the T3SS gene, the T3SS1 infection and assembly mechanism remain unclear. Only a few studies have reported that VP1656 and VP1657 act as translocators, ensuring the normal translocation of effector proteins into the host eukaryotic cytoplasm (Shimohata et al., 2012). These effectors, including VP1680, VP1683, VP1686, and VPA0450, act as virulence factors that interfere with normal signaling pathways in host cells (Yarbrough et al., 2009; Broberg et al., 2010; Sreelatha et al., 2013; Sreelatha et al., 2015). VPA0451 acts as a chaperone to stabilize VPA0450 (Waddell et al., 2014). Moreover, VP1682, as a chaperone protein of VP1680, enhances the cytotoxicity of VP1680 and is jointly regulated by small RNA spots with VP1680 (Tanabe et al., 2015).

Most gram-negative bacteria that produce T3SS polymerize a 6-kDa single needle protein of the secretion machinery into extracellular needle components that can perforate the eukaryotic plasma membrane and form a secretion conduit for effector translocation (Kimbrough and Miller, 2000; Kubori et al., 2000; Tamano et al., 2000; Blocker et al., 2001). Similar to YscF (*Y. pestis*) (Torruellas et al., 2005), PrgI (*Salmonella enterica* SPI-1) (Kato et al., 2018), and MixH (*Shigella Flexner*) (Cordes et al., 2003), the T3SS1 needle in *V. parahaemolyticus* is also critical; however, the information about it remains rather limited. In addition, effectors transported to the host eukaryotic cytoplasm by T3SS enable the subversion of host homeostasis and immune defenses (Cornelis, 2006; Galan and Wolf-Watz, 2006; Santos and Orth, 2015), thus enhancing bacterial virulence (Chung et al., 2016; Ratner et al., 2016). However, studies on the T3SS1 secretion mechanism and effector number in *V. parahaemolyticus* remain rather limited compared to those on *Y. enterocolitica*. Hence, it is essential to explore the biological functions of T3SS1 components and identify unknown effectors to elucidate the pathogenic mechanism.

In this study, we assessed the biological functions of the VscF protein, both in terms of cytotoxicity and effector translocation. Additionally, we proposed a possible role of the *vscF* gene in *V. parahaemolyticus* virulence and examined the unknown T3SS1 effector VPA0226.

## Materials and Methods

### Strains, Cell Lines, and Culture Conditions

The bacterial strains and plasmids used in this study are listed in **Table 1**. In this study, *V. parahaemolyticus* RIMD2210633 with a Δ*tdhAS* derivative was used as the wild-type (WT) POR-1 strain and cultured in Luria Bertani (LB) medium (Park et al., 2004a). *Escherichia coli* SM10λpir strains were used for the general manipulation and mobilization of plasmids into *V. parahaemolyticus via* conjugation (Miller and Mekalanos, 1988). The pMMB207 shuttle plasmid was used as an overexpression plasmid (Liu et al., 2018). Thiosulfate citrate bile salts sucrose (TCBS) agar was used to screen mutant and complementation strains containing chloramphenicol (Orkin, 1990). Antibiotics were used at the following concentrations: ampicillin, 100 μg/mL; kanamycin, 50 μg/mL; and chloramphenicol, 10 μg/mL.

**Table 1:** Plasmids and bacterial strains.

Strains or plasmids	Characteristics	Source or reference
Plasmid		
pYAK1	R6K-*ori* suicide vector containing *sacB* gene	(Kodama et al., 2002)
pYAK1-Δ*vscF*	Suicide vector pYAK-1 containing up- and downstream DNA franking sequences to the *vscF* gene for generating the *vscF* mutant	This study
pYAK1-CΔ*vscF*	Suicide vector pYAK-1 containing *vscF* gene with a single nucleotide base synonymous mutation for generating the *vscF* complementation strains	This study
pYAK1-ΔvcrD1	Suicide vector pYAK-1 containing up- and downstream DNA franking sequences to the *vcrD1* gene for generating the *vcrD1* mutant	(Park et al., 2004b)
pYAK1-ΔvcrD2	Suicide vector pYAK-1 containing up- and downstream DNA franking sequences to the *vcrD2* gene for generating the *vcrD2* mutant	(Park et al., 2004b)
pYAK1-Δvpa0226	Suicide vector pYAK-1 containing up- and downstream DNA franking sequences to the *vpa0226* gene for generating the POR-1-Δvpa0226, POR-2-Δvpa0226, and POR-3-Δvpa0226 mutants	This study
pYAK1-S151A	Suicide vector pYAK-1 containing the *vpa0226* DNA sequence with 151Ser mutating to Ala for generating the POR-1-S151A, POR-2-S151A, and POR-3-S151A mutants	This study
pMMB207	Expression vector for *V. parahaemolyticus*, Cm^r^	(Morales et al., 1991)
pMMB207-vp1683-CyaA	Derivative of pMMB207, encoding a fusion of VP1683 with the catalytic domain of adenylate cyclase (CyaA) at the C-terminus from *Bordet Ella pertussis*	This study
pMMB207-vp1686-CyaA	Derivative of pMMB207, encoding a fusion of VP1686 with the catalytic domain of adenylate cyclase (CyaA) at the C-terminus from *Bordet Ella pertussis*	This study
pMMB207-vpa0226-CyaA	Derivative of pMMB207. encoding a fusion of VPA0226 with the catalytic domain of adenylate cyclase (CyaA) at the C-terminus from *Bordet Ella pertussis*	This study
Strain		
*E. coli*		
SM10λpir	thi thr leu tonA lacy supE recA:RP4-2-Tc: Mu λpir, OriT of RP4 Kmr; conjugational donor	(Miller and Mekalanos, 1988)
SM10λpir/1683CyaA	SM10λpir containing plasmid pMMB207- vp1683-CyaA	This study
SM10λpir/1686CyaA	SM10 λpir containing plasmid pMMB207- vp1686-CyaA	This study
SM10λpir/0226CyaA	SM10 λpir containing plasmid pMMB207- vpa0226-CyaA	This study
*V. parahaemolyticus*		
KXV237	RIMD 2210633(KP positive, serotype O3:K6)	(Makino et al., 2003)
POR-1	ΔtdhAS derivative of KXV237	(Park et al., 2004a)
POR-1/VP1683-CyaA	POR-1 containing plasmid pMMB207- vp1683-CyaA	This study
POR-1/VP1686-CyaA	POR-1 containing plasmid pMMB207-vp1686-CyaA	This study
POR-1/VPA0226-CyaA	POR-1 containing plasmid pMMB207-vpa0226-CyaA	This study
POR-1-Δvpa0226	POR-1 knockout of *vpa0226*	This study
POR-1-S151A	POR-1-Δvpa0226 complementation strains with mutant VPA0226 S151A	This study
POR-2	*vcrD1* knockout of POR-1(ΔT3SS1 strain)	(Park et al., 2004b)
POR-2/VPA0226CyaA	POR-2 containing pMMB207-vpa0226-CyaA	This study
POR-2/VP1683-CyaA	POR-1 containing plasmid pMMB207- vp1683-CyaA	This study
POR-2/VP1686-CyaA	POR-1 containing plasmid pMMB207-vp1686-CyaA	This study
POR-2-Δvpa0226	POR-2 knockout of *vpa0226*	This study
POR-2-S151A	POR-2-Δvpa0226 complementation strains with mutant VPA0226 S151A	This study
POR-3	*vcrD2* knockout of POR-1 (ΔT3SS2 strain)	(Park et al., 2004b)
POR-3/VP1683-CyaA	POR-1 containing plasmid pMMB207- vp1683-CyaA	This study
POR-3/VP1686-CyaA	POR-1 containing plasmid pMMB207-vp1686-CyaA	This study
POR-3/VPA0226CyaA	POR-3 containing pMMB207-vpa0226-CyaA	This study
POR-3-Δvpa0226	POR-3 knockout of *vpa0226*	This study
POR-3-S151A	POR-3-Δvpa0226 complementation strains with mutant VPA0226 S151A	This study
ΔvcrD1/ΔvcrD2	*vcrD1*/*vcrD2* knockout of POR-1 (ΔT3SS1/ΔT3SS2 strain)	(Kodama et al., 2007)
ΔvcrD1/ΔvcrD2/VPA0226CyaA	ΔvcrD1/ΔvcrD2 containing plasmid pMMB207-vpa0226-CyaA	This study
ΔvscF	POR-1 knockout of *vscF* (deletion from nt 28 to 250 of the gene)	This study
Δ*vscF*/VP1683-CyaA	Δ*vscF* containing pMMB207- vp1683-CyaA	This study
Δ*vscF*/VP1686-CyaA	Δ*vscF* containing pMMB207-vp1686-CyaA	This study
ΔvscF/VPA0226-CyaA	Δ*vscF* containing pMMB207-vp1686-CyaA	This study
CΔ*vscF*	*vscF* complementation strains	This study
CΔvscF/VP1683-CyaA	CΔ*vscF* containing pMMB207- vp1683-CyaA	This study
CΔ*vscF*/VP1686-CyaA	CΔ*vscF* containing pMMB207-vp1686-CyaA	This study
C△*vscF*/VPA0226-CyaA	CΔ*vscF* containing pMMB207-vp1686-CyaA	This study
Δ*vscI*	POR-1 knockout of *vscI*	This study
Δ*vscI*/VPA0226-CyaA	Δ*vscI* containing pMMB207-vp1686-CyaA	This study
CΔ*vscI*	*vscI* complementation strains	This study
CΔvscI/VPA0226-CyaA	CΔ*vscI* containing pMMB207-vp1686-CyaA	This study

HeLa, Raw264.7, and Caco-2 cells were cultured in Dulbecco’s modified Eagle’s medium (DMEM) (Invitrogen) supplemented with 10% heat-inactivated fetal bovine serum (FBS; Invitrogen).

### Construction of Mutants

The technology for the construction of mutant strains was based on a previous report (Park et al., 2004b). Briefly, overlap PCR was used to amplify DNA fragments containing the in-frame deletion mutant *vscF* using appropriate primers, as listed in **Table 2**. The DNA fragments were then cloned into the suicide pYAK1 vector to construct pYAK1-Δ*vscF* plasmids, which were introduced into POR-1 strains to construct the mutant Δ*vscF*.

**Table 2:** Sequences of the primers used for the construction of mutants and expression vectors.

Primer	Sequence (5′ to 3′)
Used for gene deletion	
vscF-1	CAGGTCGACTCTAGAGGATCCGGGTTATCGCATATCCAG
vscF-2	CGTTCCATGCACTGTTGGTCGCATCGTA
vscF-3	GACCAACAGTGCATGGAACGAGAATTAC
vscF-4	CGAGCTCGGTACCCGGGGATCCTTCCTAATGTCTTGGGTG
vpa0226-1	CAGGTCGACTCTAGAGGATCCTGACCGTGATGCCAAAAT
vpa0226-2	AGCCGTGTCTGCGATACCAACAGCGAAC
vpa0226-3	TTGGTATCGCAGACACGGCTTCTGAGTT
vpa0226-4	CGAGCTCGGTACCCGGGGATCCAATGCCTTGTCGGCACGA
Used for the construction of complementation strains	
C-vscF-1	CAGGTCGACTCTAGAGGATCCGGGTTATCGCATATCCAG
C-vscF-2	CGAGGTTTACACTGTTGGTCGCATCATA
C-vscF-3	GACCAACAGTGTAAACCTCGATGACGTTAA
C-vscF-4	CGAGCTCGGTACCCGGGGATCCTTCCTAATGTCTTGGGTG
S151A-1	CAGGTCGACTCTAGAGGATCCGGGATGATGAAAAAAACAATCACAC
S151A-2	GTATCAGACAATGCGTCACCGAGTGCAACC
S151A-3	GGTGACGCATTGTCTGATACAGGCAACATC
S151A-4	CGAGCTCGGTACCCGGGGATCCAATGCCTTGTCGGCACGAGA
Used for the construction of expression vectors	
pM207-vp1683CyaA1	GAGCTCGGTACCCGGGGATCCCATGGTTAATATCAATACGTC
pM207-vp1683CyaA2	GCGATTGCTGACCAAGTTTGTGGCTAT
pM207-vp1683CyaA3	CAAACTTGGTCAGCAATCGCATCAGGCTGG
pM207-vp1683CyaA4	CAGGTCGACTCTAGAGGATCCTTATGTCATAGCCGGAATCC
pM207-vp1686CyaA1	GAGCTCGGTACCCGGGGATCCCATGATCAGTTTTGGAAATGT
pM207-vp1686CyaA2	GCGATTGCTGTTTGATACCGTGAAGGCTAT
pM207-vp1686CyaA3	CGGTATCAAACAGCAATCGCATCAGGCTGG
pM207-vp1686CyaA4	CAGGTCGACTCTAGAGGATCCTTATGTCATA GCCGGAATCC
pM207-vpa0226CyaA1	GAGCTCGGTACCCGGGGATCCCATGATGAAAAAAACAATCAC
pM207-vpa0226CyaA2	GCGATTGCTGGAAACGGTACTCGGCTAAGT
pM207-vpa0226CyaA3	GTACCGTTTCCAGCAATCGCATCAGGCTGG
pM207-vpa0226CyaA4	CAGGTCGACTCTAGAGGATCCTTATGTCATA GCCGGAATCC
Used for qRT-PCR	
16sRNA-F	GACACGGTCCAGACTCCTAC
16sRNA-R	GGTGCTTCTTCTGTCGCTAAC
vpa0226RT-F	CAAACCAGCAAACACCTT
vpa0226RT-R	GTCCGTCAAACGAATCAG

Underscore served as indicate vector upstream or downstream sequence.

Furthermore, complementation strain CΔ*vscF* was constructed using genetic complementation by inserting the *vscF* gene with a synonymous point mutation into the genome of the Δ*vscF* mutant. The *vscF* gene and its two flanking regions were amplified from the POR-1 genomic DNA, allowing a point mutation (T4A) in *vscF*. CΔ*vscF* was screened and identified using a method similar to that used for the Δ*vscF* construction.

The same technology was used to generate the POR-1-Δvpa0226, POR-2-Δvpa0226, and POR-3-Δvpa0226 mutants, as well as POR-1-/POR-2-/POR-3-S151A.

### Growth Curve

The growth curve was examined by turbidity measurements at 600 nm (OD600) using LB broth as previously described (Huang et al., 2019). Overnight-cultured WT POR-1, Δ*vscF*, and CΔ*vscF* were transferred into 50 mL LB broth (volume ratio: 1:100) and cultured at 37°C with shaking at 220 rpm. Cultures were measured, and the OD600 values were recorded every 1 h. At least three independent experiments were conducted. GraphPad Prism was used to draw the growth curve and perform statistical analysis using one-way analysis of variance (ANOVA).

### Cytotoxicity Assay

Cytotoxicity was assessed *via* morphology and the release of lactate dehydrogenase (LDH) using an LDH Cytotoxicity Assay Kit (Beyotime, C0016) (Liu et al., 2018). First, HeLa cells were infected with WT POR-1, Δ*vscF*, and CΔ*vscF* for 3 h with 5% CO_2_ at a multiplicity of infection (MOI) of 10 in DMEM supplemented with 1% FBS (Kodama et al., 2007), washed three times with sterile phosphate buffered saline (PBS), fixed with 4% paraformaldehyde, and then permeabilized with 0.1% Triton X-100. Samples were incubated with Tubulin-Tracker Red (Beyotime, diluted 1:50 with 5% BSA) at room temperature (25°C) for 1 h, and then counterstained with DAPI at 37°C for 15 min. Finally, the samples were washed three times with sterile PBS, and cell morphology changes were observed in the experimental groups using a ZEISS Oxi0 Observer Inverted microscope.

HeLa, Caco-2, and Raw264.7 cells were infected with WT POR-1, Δ*vscF*, and CΔ*vscF* using the above-mentioned method in sterile micro 96-well plates for 3 h at 37°C with 5% CO_2_. The release of LDH into the medium was quantified using the LDH Cytotoxicity Assay Kit according to the first method in the manufacturer’s instructions. Uninfected cells and cell-free well plates were used as negative and background controls, respectively. The LDH released by the lysis of cells with 10% (vol/vol) LDH release reagent was defined as the maximum cell release. The release of LDH was measured at OD490, which subtracted the absorbance of the background control group, which was the actual value. Cytotoxicity was calculated as follows: % cytotoxicity (test LDH release—cell spontaneous release—bacteria spontaneous release)/(cell maximal release—cell spontaneous release). Three independent experiments were conducted.

The release of LDH was quantified using the same methods used for the quantification of vpa0226-related diverse mutants. At least three independent experiments were conducted.

### Adhesion Assay

The adhesion ability of the WT and mutant strains was determined using HeLa cells. Strains were grown in LB at 37°C to logarithmic phase, harvested by centrifugation at 5000 g for 10 min at low temperature, washed three times with pre-cooled PBS, and suspended in DMEM supplemented with 1% FBS. HeLa cells were grown in sterilized 12-well plates with DMEM supplemented with 10% FBS to 80%–90%. The preprocessed bacterial suspension was used to replace the DMEM in 12-well plates, at a final MOI of 10, and then incubated at 37°C with 5% CO_2_ in an incubator for 2 h. Subsequently, the non-adherent bacteria were removed by washing three times with PBS. HeLa cells were then lysed with 200 μL per well of 0.1% Triton X-100 (v/v) for 10 min. Bacterial cell counts were determined by serially diluting the samples on the LB medium plates. At least three independent experiments were conducted.

### Detection of Apoptosis and Mortality of Cells

Due to caspase-3-induced apoptosis (Serapio-Palacios and Navarro-Garcia, 2016), in this study, the GreenNuc™ caspase-3 assay kit (Beyotime, C1168S) and propidium iodide (PI) were used to detect apoptotic cells and cells with damaged membranes, respectively. With the same infection method as adhesion assay, after infection, the cells were digested with 0.25% trypsin for 3–5 min, collected by centrifugation at 600 × g for 5 min and washed three times with sterile PBS. The sample cells were incubated with GreenNuc™ caspase-3 (5 μM) and PI (10 μmol/L) at room temperature (25°C) for 30 min and kept away from light. Fluorescence-activated cell sorting was used to screen and detect the fluorescent cells. Sterile PBS was added to the wells to infect HeLa cells as a blank control. At least three independent experiments were performed, and GraphPad Prism was used to perform the statistical analyses.

### Construction of the Adenylate Cyclase Reporter

In 1994, the adenylate cyclase reporter vector system was first developed by Sory et al. to detect the translocation of prokaryotic bacterial effectors in eukaryotic cells (Sory and Cornelis, 1994). Subsequently, this system has been used in a variety of bacteria, including *V. parahaemolyticus* (Shimohata et al., 2012; Chakravarthy et al., 2017). In fact, it uses a fusion protein of the target effector protein and *Bordetella pertussis* adenylate cyclase toxin (CyaA) to complete its purpose. In this study, we used this system to determine whether the identified secreted proteins were translocated into eukaryotic cells. Initially, we amplified the DNA sequences vp1683, vp1686, vpa0226, and cyaA using appropriate primers, as listed in **Table 2**. We then constructed fusion DNA sequences *via* overlap PCR using the appropriate primers, as listed in **Table 2**. Ultimately, fusion sequences were cloned into the pMMB207 vector to construct pMMB207-vp1683-CyaA, pMMB207-vp1686-CyaA, and pMMB207-vpa0226-CyaA fusion protein expression plasmids using the One Step Cloning Kit (Vazyme, C112).

### Quantitative Reverse Transcription-PCR

The transcription level of vpa0226-cyaA fusion sequence in bacteria was determined by quantitative reverse transcription-PCR (qRT-PCR). POR-1, Δ*vscF*, and CΔ*vscF* containing pMMB207-vpa0226-CyaA overexpression plasmid were grown in LB medium at 37°C to logarithmic phase, and RNA was extracted using an E.Z.N.A. bacterial RNA kit (Omega, USA). The mRNA transcription levels of vpa0226 were examined using Step-One and Step-One Plus Real-Time PCR machine (Thermo Fisher) with TB Green (Takara) using appropriate primers, as listed in **Table 2**. Using 16sRNA as an internal reference for normalization, the relative expression ratio was calculated for vpa0226 gene using the delta–delta threshold cycle (Ct) method (Pfaffl, 2001).

### Measurement of Cyclic AMP (cAMP) Production and Western Blot Analysis

The adenylate cyclase reporter plasmids with fusion secreted effectors (pMMB207-vp1683-CyaA, pMMB207-vp1686-CyaA, and pMMB207-vpa0226-CyaA) were introduced into POR-1, Δ*vscF*, and CΔ*vscF* strains to fabricate expression strains. Owing to cytotoxicity up to 90% and cell lysis for 3 h, we used the expression strains to infect HeLa cells for 2 h to identify intracellular cAMP levels using a cAMP ELISA product kit (R&D Systems, USA.) Following the manufacturer’s instructions (Kodama et al., 2007), and western blot analysis was performed using CyaA antibody (Santa Cruz) (Ono et al., 2006; Shimohata et al., 2012). Densitometric analyses were performed using the ImageJ software.

Additionally, the pMMB207-vpa0226-CyaA plasmid was introduced into POR-1, Δ*vcrD1* (POR-2), Δ*vcrD2* (POR-3), and Δ*vcrD1*/Δ*vcrD2* strains to determine translocations (Park et al., 2004b). The pMMB207-vp1683-CyaA and pMMB207-vp1686-CyaA plasmids served as positive controls. The same processes were employed to examine whether VPA0226 translocates into the HeLa eukaryotic cytoplasm *via* T3SS1. Densitometric analyses were performed using the ImageJ software.

### Secretion Assay of VPA0226

The secretion assay of effector proteins was based on a previous report (Chimalapati et al., 2020) and made a slight change. Overnight grown *V. parahaemolyticus* POR-1, POR-2, POR-3, and Δ*vcrD1*/Δ*vcrD2* strains containing pMMB207-vpa0226-CyaA plasmid in MLB at 30°C were diluted to an OD600 of 0.3 in LB broth, grown at 37°C for 3 h to an OD600 of 0.5. Bacterial cultures were collected by centrifugation at 5000× g for 5 min, washed three times with sterile PBS, and then resuspended in 1× PBS. The supernatants of bacterial culture were filtered with a 0.22-micron filter and concentrated with an ultrafiltration tube (aperture: 30kD) by centrifugation at 3000× g for 20 min at 4°C. After centrifugation, the membrane was washed with 1× PBS buffer to obtain the concentrated protein. The secretion amounts of VPA0226 were analyzed by western blotting. Densitometric analyses were performed using the ImageJ software.

### Statistical Analyses

Statistical analyses were performed using the GraphPad Prism software. The t-test was used to analyze the differences between experimental groups. Statistical significance was set at *P* < 0.05.

## Results

### VscF Is Required for *V. parahaemolyticus* Cytotoxicity and Adhesion

**Figure 1:**
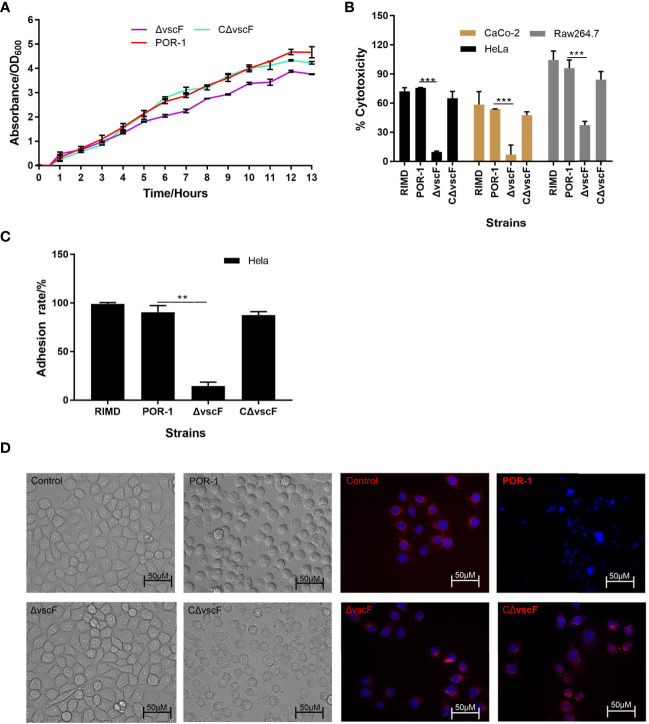
The biological characteristics of WT POR-1, Δ*vscF* mutant, and complementation CΔ*vscF* strains. Growth curves **(A)** of *V. parahaemolyticus* strains were determined with the deletion of the *vscF* gene. Cytotoxic **(B)** and adherent **(C)** abilities of *V. parahaemolyticus* mutant strains were examined using infected HeLa cells. Values are presented as means and standard deviations from three biological replicates. **P < 0.01 and ***P < 0.001. HeLa morphology under co-incubation with *V. parahaemolyticus* strains was observed using an inverted microscope. DNA was stained with DAPI (blue), whereas the cell skeleton was stained with Tubulin-Tracker (red) **(D)**.

We determined whether *vscF* participates in regulating growth, virulence, cytotoxicity, and adhesion. As shown in **Figure 1**, the trend and speed of growth were not notably different (**Figure 1A**), whereas cytotoxicity and adhesion significantly decreased (**Figures 1B, C**) with *vscF* deletion. Moreover, under phase-contrast microscopy, HeLa morphology of the Δ*vscF* group contained only a few rounded cells that tended to the control group compared with those in the POR-infected group. Meanwhile, the red tubulin skeleton of the Δ*vscF* group was distributed in a radial pattern. HeLa cells again possessed a round shape, and red fluorescence gathered around the nucleus when *vscF* was complemented (**Figure 1D**).

### VscF Is Involved in the Modulation of Host Apoptosis

**Figure 2:**
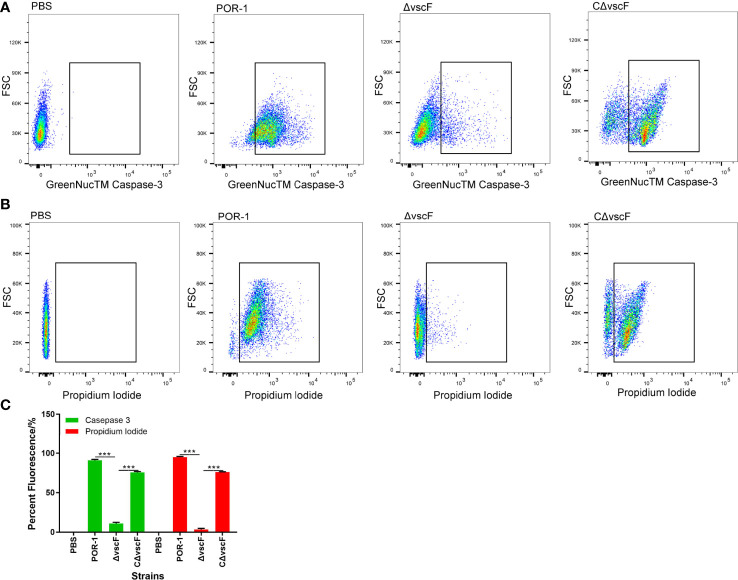
Detection of HeLa cell apoptosis caused by *V. parahaemolyticus* strains using GreenNucTM Caspase-3 Assay Kit and propidium iodide. Fluorescence-activated cell sorting was used to detect fluorescence **(A, B)**, and PBS was used as the blank (control). Values are the means and standard deviations from three biological replicates. Percentage represents the portion of the fluorescently-stained cells in a total of certain number of assessed HeLa cells **(C)**. ***P < 0.001.

To examine whether *vscF* has an influence on host cell apoptosis and cytotoxicity, caspase-3 and PI were used to detect apoptosis of host cells in the POR-1, Δ*vscF*, and CΔ*vscF* infection groups. As shown in **Figures 2A, B**, the fluorescence of caspase-3 staining significantly decreased (*P* < 0.01, **Figure 2C**) from 91.9% to 10.1% in the Δ*vscF* group, which was supplemented to 75.2% in the CΔ*vscF* group. Meanwhile, the fluorescence of PI staining significantly decreased (*P* < 0.01, **Figure 2C**) from 95.7% to 2.3% in the Δ*vscF* group, which was supplemented to 76.5% in the CΔ*vscF* group. These results suggested that *vscF* is involved in the apoptosis of HeLa cells.

### VscF, Named Needle, Controls T3SS1 Effector Translocation

**Figure 3:**
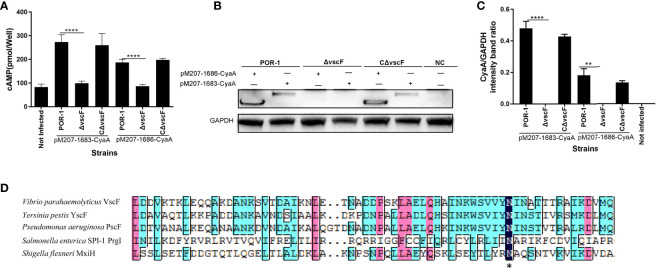
The *vscF*, encoding needle protein, is diversely involved in the secretion of effector proteins in *V. parahaemolyticus*. Intracellular cAMP **(A)** and the translocated amounts of CyaA-fused protein **(B)** were quantified after infection with *V. parahaemolyticus* strains. GAPDH served as internal reference. “+” or “-” refers to a strain harboring pMMB207-vp1683/1686-CyaA expression vector (+) or not (-), NC served as cell control group. Densitometry analysis was performed for relative quantification **(C)**. ****P < 0.0001, **P < 0.01, and *P < 0.05. Sequence alignments of *vscF* were performed using the DNAMAN program. Dark blue boxes and asterisk represents 100% conserved residues; pink boxes represent 80% conserved residues; light blue boxes represent 60% conserved residues **(D)**.

To study whether *vscF* affects effector translocation, a translocation assay was performed using the adenylate cyclase reporter vector system. As shown in **Figure 3**, the cAMP ELISA product kit data showed that the intracellular cAMP level in the Δ*vscF* group was significantly lower than that of the POR-1 and CΔ*vscF* groups (*P* < 0.01; **Figure 3A**). In addition, the results of western blotting showed that the intracellular level of CyaA protein decreased in the Δ*vscF*-infected HeLa group (**Figure 3B**) compared with that in the POR-1-infected group, which was consistent with the cAMP results. Densitometry and statistical analysis showed significant differences in the intracellular levels of CyaA protein between the POR-1- and Δ*vscF*-infected groups (**Figure 3C**).

In addition, as shown in **Figure 3D**, VscF aligned with other gram-negative bacteria T3SS needle proteins, and the transmembrane structure of VscF was predicted contain 1–23 signal peptides in N-Terminus using Phyre2. These results suggested that the *vscF* gene may encode needle proteins and that its deletion can prevent the translocation of VP1683 and VP1686 from bacteria into HeLa cells.

### Bacterial Protein VPA0226 Translocates Into the Host Eukaryotic Cytoplasm, Probably *via* T3SS1, Whose Translocation May Be Manipulated by T3SS1 VscF and VscI

**Figure 4:**
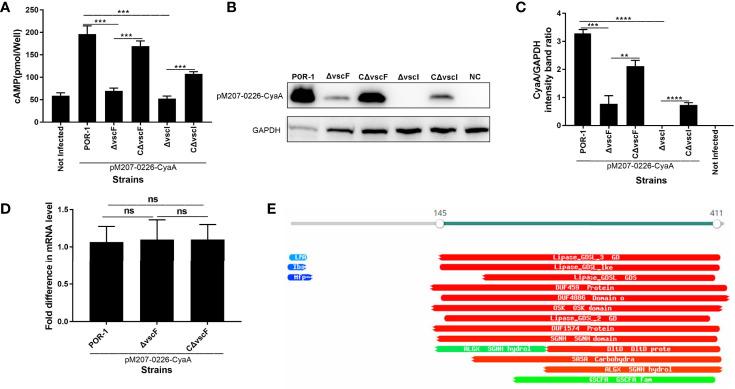
*vscF* and *vscI* are involved in the secretion of VPA0226 in *V. parahaemolyticus*. Intracellular cAMP **(A)** and the translocated amounts of CyaA-fused protein **(B)** were quantified after infection with *V. parahaemolyticus vscF* and *vscI* mutant strains. GAPDH served as internal reference. “+” or “-” refers to a strain harboring pMMB207-vpa0226-CyaA expression vector (+) or not (-). Densitometry analysis was performed for relative quantification **(C)**. ****P < 0.0001, ***P < 0.001, and **P < 0.01. Relative mRNA transcription levels of vpa0226 genes in POR-1, Δ*vscF* and CΔ*vscF* strains. For each sample, the acquired cycle threshold (CT) was normalized to the CT of the internal housekeeping gene 16s RNA, and the ΔCT was normalized to the ΔCT of the POR-1 strain. Relative fold differences in mRNA expression level were calculated using the 2^−ΔΔCT^ method **(D)**. Schematic illustration of the conserved domains of the predicted effector encoded by vpa0226. The predicted domains of VPA0226 harboring the typical GDLS lipase are indicated **(E)**. ns, indicates no significant difference.

To investigate whether VPA0226 translocates into the host cell cytoplasm *via* T3SS1, a translocation assay was performed using the same methods as described above. As shown in **Figure 4**, the cAMP ELISA product kit data showed that the intracellular cAMP levels of the Δ*vscF* and Δ*vscI* groups were significantly lower than those of the POR-1, CΔ*vscF*, and CΔ*vscI* groups (*P* < 0.01; **Figure 4A**). Further, western blotting results showed that the translocated amounts of CyaA-fused protein decreased in the Δ*vscF* and Δ*vscI* groups (**Figure 4B**). Additionally, densitometry and statistical analysis showed that the decrease in the translocated amounts of CyaA-fused protein (**Figure 4B**) were significantly different (*P* < 0.01; **Figure 4C**).

To study whether the transcription level of vpa0226 leads to the above significant difference, qRT-PCR was performed and the results showed that there was no significant difference between POR-1, Δ*vscF*, and CΔ*vscF* groups in vpa0226 mRNA level (**Figure 4D**). Therefore, the deletion of *vscF* affected the secretion of VPA0226 rather than the expression.

A protein motif search showed that VPA0226 might harbor GDSL (SGNH) hydrolases and several putative enzyme domains (**Figure 4E**). Thus, these data suggested that both *vscF* and *vscI* possess the ability to control VPA0226 translocation; that is, VPA0226 secretion may occur through T3SS1.

### VPA0226 Secreted Protein Can Be Secreted *via* T3SS1 and Translocated Into the Host Eukaryotic Cytoplasm *via* T3SS1

**Figure 5:**
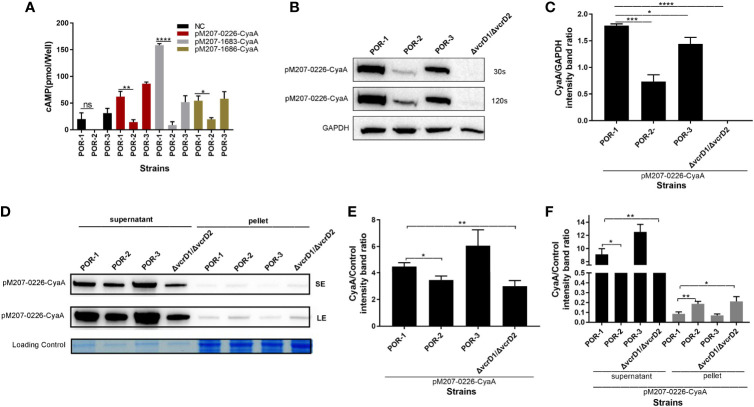
VPA0226 is secreted by T3SS1. Intracellular cAMP levels were quantified after infection with *V. parahaemolyticus* POR-1, POR-2, and POR-3 strains, which harbored pMMB207-vpa0226-CyaA, pMMB207-vp1683-CyaA, and pMMB207-vp1686-CyaA, respectively **(A)**. Intracellular translocated amounts of CyaA-fused protein were also examined after infection with *V. parahaemolyticus* POR-1, POR-2, and POR-*3* strains harboring the pMMB207-vpa0226-CyaA expression vector. GAPDH served as internal reference. “+” or “-” refers to a strain harboring pMMB207-vpa0226-CyaA expression vector (+) or not (-) **(B)**. Densitometry analysis of B was performed for relative quantification **(C)**. Secretion of VPA0226 (supernatant) from POR-1, POR-2, POR-3 and Δ*vcrD1*/*vcrD2* strains harboring the pMMB207-vpa0226-CyaA vector were detected by immunoblotting with anti-CyaA antibody **(D)**. Loading control served as total bacterial lysate or total secretion media, LE, Long exposure; SE, Short exposure. Densitometry analysis of SE and LE were respectively performed for relative quantification, as shown in **(E, F)** E (the supernatant of SE): the CyaA/Control (intensity band ratio) of POR-1, POR-2, POR-3 and Δ*vcrD1*/*vcrD2* is 4.47 (60725/13565), 3.51 (43948/12494), 6.00 (77525/12919), and 2.98 (30288/10145), respectively. F (the supernatant of LE): the CyaA/Control (intensity band ratio) of POR-1, POR-2, POR-3 and Δ*vcrD1*/*vcrD2* is 9.11 (123677/13565), 7.39 (92309/12494), 12.48 (161263/12919), and 6.54 (66395/10145), respectively. F (the pellet of LE): the CyaA/Control (intensity band ratio) of POR-1, POR-2, POR-3 and Δ*vcrD1*/*vcrD2* is 0.09 (2362/27281), 0.18 (6382/34193), 0.07 (1788/25424) and 0.21 (5996/28786), respectively. ****P < 0.0001, ***P < 0.001, **P < 0.01, and *P < 0.05.

To confirm that VPA0226 is transported to the host eukaryotic cytoplasm using T3SS1, although it is located on chromosome 2, a translocation assay was performed using traditional POR-1, POR-2, POR-3, and Δ*vcrD1*/Δ*vcrD2* strains (Park et al., 2004b). As shown in **Figure 5**, the intracellular cAMP level was significantly lower in the POR-2 infection group than in the POR-1 and POR-3 infection groups (*P* < 0.01; **Figure 5A**), while the translocated amounts of VPA0226 decreased with ECL™ Prime western blotting detection (GE Healthcare) at 30 s and 120 s (**Figure 5B**), and densitometry and statistical analysis showed significant differences (*P* < 0.01; **Figure 5C**). The secretion assay of VPA0226 was performed using various *V. parahaemolyticus* strains, including POR-1, POR-2, POR-3, and Δ*vcrD1*/Δ*vcrD2* strains containing pMMB207-vpa0226-CyaA plasmid. We observed that VPA0226 secretion (supernatant) was significantly decreased in POR-2 and Δ*vcrD1*/Δ*vcrD2* compared with POR-1 group, while the pellet detection level of VPA0226 in POR-2 and Δ*vcrD1*/Δ*vcrD2* was significantly increased (*P* < 0.01; **Figures 5D–F**). Therefore, these results demonstrated that the secreted protein VPA0226 can be secreted by T3SS1, and is translocated into the host eukaryotic cytoplasm by T3SS1 when *V. parahaemolyticus* infects eukaryotic host cells.

**Figure 6:**
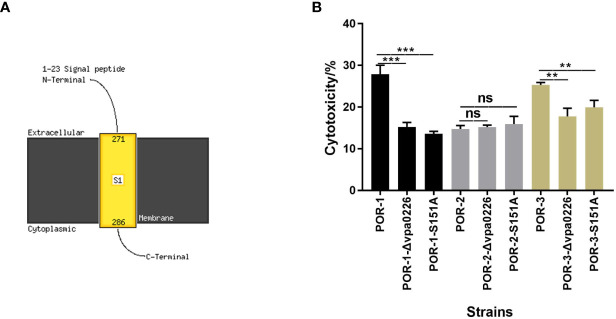
S151 of VPA0226 was a significant active site. The transmembrane helices of VPA0226 were predicted to contain 1–23 signal peptides in N-Terminus using Phyre2 **(A)**. The cytotoxicity of *V. parahaemolyticus vpa0226*-related mutant strains were examined using infected HeLa cells **(B)**. ***P < 0.001, **P < 0.01. ns, indicates no significant difference.

In addition, transmembrane helix prediction indicated that the N-terminus of VPA0226 contains 1–23 signal peptides, highlighting that VPA0226 can be secreted from the bacterial cytoplasm to the extracellular space (**Figure 6A**).

To identify the essential functional sites in VPA0226, the cytotoxicity levels of the diverse mutants were determined. As shown in **Figure 6B**, both the inactivation and point mutation (S151A) of VPA0226 reduced its cytotoxicity, confirming that Ser-151 is an important functional site (**Figure 6B**).

## Discussion

*V. parahaemolyticus* is an important foodborne pathogen that is naturally found in marine environments, including estuaries (Broberg et al., 2011). T3SS1 is an essential bacterial survival mechanism that induces toxicity in eukaryotic cells (Park et al., 2004b). In this study, we constructed the inactivated and complemented strains of the *vscF* (vp1694) gene and showed that Δ*vscF* exhibited reduced cytotoxicity (**Figure 1B**), indicating that *vscF* is directly or indirectly involved in the regulation of bacterial virulence and plays a critical role in T3SS1.

In most pathogens, adhesion is an important virulence factor closely related to pathogenicity and is considered the first step in the pathogenesis of microbial infections (Stones and Krachler, 2016). Therefore, we examined whether *vscF* influences adhesion. Compared with WT POR-1, the mutant strain showed significantly reduced adhesion ability (**Figure 1C**), indicating that T3SS1 is also tightly associated with adhesion ability. In 1989, Yamamoto and Yokota (1989), (Jiang et al., 2014) demonstrated an association between type VI secretion system 2 and adhesion. Compared with T3SS2, a study by Zhou et al. (2014) showed that VopV can assist *V. parahaemolyticus* in adhering to host cells *via* “effacement” of microvilli. However, we did not study the mechanism by which T3SS1 or *vscF* mediates adhesion; only the adhesion phenotype was studied.

Apoptosis plays an important role in the progression of bacterial infections (Peters et al., 2013). Studies have reported that the death of HCT116 and HeLa cells caused by T3SS1 is actually apoptosis (Bhattacharjee et al., 2005; Ono et al., 2006), and Bi et al. reported that in *Y. pestis* T3SS, reduced apoptosis is related to the caspase-3 signaling pathway (Bi et al., 2009). In this study, we observed that the apoptosis of HeLa host cells in the Δ*vscF*-infected group was significantly lower than that in the POR-1- and CΔ*vscF*-infected groups (**Figure 2**), indicating that *vscF* is involved in the apoptosis of HeLa cells.

T3SS introduces a range of effectors into host eukaryotic cells through a syringe-like transmembrane device (Shames and Finlay, 2012; Portaliou et al., 2016), and these effectors participate in manipulating the host protein function or signaling pathways to exert virulence (Bhattacharjee et al., 2006; Burdette et al., 2009; Yarbrough et al., 2009; Higa et al., 2013). Therefore, effector translocation is vital for bacterial infectivity, which was the focus of this study. Thus, we examined the translocation of prokaryotic *V. parahaemolyticus* effectors to eukaryotic cells using an adenylate cyclase reporter vector system (Sory and Cornelis, 1994). We observed that the intracellular levels of VP1683 and VP1686 proteins notably decreased in the Δ*vscF* group compared to those in the POR-1 and CΔ*vscF* groups (**Figures 3A–C**), indicating that *vscF* is necessary for the assembly of the T3SS1 translocation device and translocation of effectors. In most gram-negative pathogens, T3SS is assembled from various proteins, and effectors can be transported to the host eukaryotic cytoplasm only when the structural components are highly coordinated (Blocker et al., 2008). Surprisingly, VscF is homologous to MixH needle proteins using DANMAN (**Figure 3D**). Needles of T3SS are the core structure of extracellular needle complexes that are responsible for the transport of effectors (Dey et al., 2019). Hoiczyk and Blobel (2001), Kusmierek et al. (2019) have demonstrated that, *Y. enterocolitica* polymerizes a single needle protein into needle punctures in eukaryotic cells.

To date, only four *V. parahaemolyticus* effectors have been reported, including VP1680, VP1683, VP1686, and VPA0450 (Bhattacharjee et al., 2006; Burdette et al., 2009; Yarbrough et al., 2009; Higa et al., 2013; Waddell et al., 2014). Based on our previous transcriptomics analysis (PRJNA601057) and the literature, we speculated that VPA0226 belongs to the T3SS effector protein of *V. parahaemolyticus*. In fact, a study on VPA0226 by Chimalapati et al. was recently first reported and has demonstrated that VPA0226, a constitutively secreted lipase, through T2SS secretion not T3SS, is required for the escape of *V. parahaemolyticus* from host cells (Chimalapati et al., 2020). The supernatant amounts of vpa0226 in the CAB2 and CAB4 strains was reduced compared to CAB3, but this difference was not quantified (like grayscale analysis) in the study of Chimalapati et al. So, we again studied the relationship between VPA0226 and T3SS1, and speculated that VPA0226 maybe also be secreted by T3SS1, except for T2SS. In addition, VPA0226 was considered to be homologous to the T3SS effector SseJ of *Salmonella*, although it is located on chromosome 2 of *V. parahaemolyticus* (Christen et al., 2009; Kolodziejek and Miller, 2015). Like VPA0450, it has been reported that it can be secreted by T3SS1, although it is located on chromosome 2. In our previous study, *vscI* was found to be critical for effector translocation. In the present study, our results demonstrated that VPA0226 was translocated in HeLa eukaryotic cytoplasm, and its translocated amounts significantly decreased in the Δ*vscF* and Δ*vscI* infection groups compared with those in the POR-1 and CΔ*vscF* or CΔ*vscI* infection groups (**Figure 4**). Hence, similar to VP1683 and VP1686, VPA0226 could be transported to host cells *via* T3SS1. qRT-PCR results showed that the mRNA level of vpa0226 had no difference between POR-1, CΔ*vscF* and CΔ*vscF* strains, which revealed that T3SS1 indeed can affected the translocation of VPA0226 (**Figure 4D**).

We further determined VPA0226 translocation rates and showed that the rates of POR-2 and Δ*vcrD1*/*vcrD2* were significantly lower than those in POR-1 and POR-3 (**Figures 5A–C**), indicating that VPA0226 is closely related to T3SS1 and not T3SS2. Additionally, the data of secretion assay showed that the secretion amounts (supernatant) of VPA0226 in POR-1 was significantly higher than those in POR-2 and Δ*vcrD1*/Δ*vcrD1*. On the contrary, the VPA0226 protein in pellet of POR-2 and Δ*vcrD1*/*vcrD2* were significantly higher than those in POR-1 (**Figures 5D–F**), which indicated that although the deletion of T3SS1 (POR-2) did not make it entirely lose the secretion of VPA0226, but the secretion amounts of VPA0226 was significantly reduced, indicating that the deletion of T3SS affects the secretion of VPA0226. Thus, we speculate that VPA0226 might be secreted into the host eukaryotic cytoplasm through T3SS1 and T2SS in *V. parahaemolyticus*. This result is not surprising considering what is known about the redundant mechanisms used by pathogens. For instance, Matsuda et al. (2019) discussed the export of a *V. parahaemolyticus* toxin by Sec and type III secretion machineries in tandem.

In summary, our results revealed that the *vscF* gene has vital effects on the virulence-associated traits of *V. parahaemolyticus*. The results of the phenotypic assays and sequence alignment suggested that the *vscF* gene encodes the needle protein of T3SS1 in *V. parahaemolyticus*. Moreover, VPA0226 was identified as an unpublished T3SS1 effector, and future studies on VPA0226 function are expected to provide insights into the mechanism by which *V. parahaemolyticus* causes a disease.

## Data Availability Statement

The original contributions presented in the study are included in the article/supplementary material. Further inquiries can be directed to the corresponding author.

## Author Contributions

LL: conceptualization, writing original draft and formal analysis. JX and WL: software and resources. JR, JD, and FT: project administration. FX: funding acquisition. All authors contributed to the article and approved the submitted version.

## Funding

This study was funded by the National Natural Science Foundation of China (31871893), the National Key Research and Development Program of China (2017YFF0208600), Jiangsu Agricultural Independent Innovation Project (SCX (18)2011), The National “Youth Top-notch Talent” Support Program (W0270187), Introduction of Nanjing Agricultural University Scientific Research Grants Project (804121), Central Guidance for Local Science and Technology Development (No. YDZX20173100004528), Science and Technology Joint Project of the Yangtze River Delta (No.17395810102), and Jiangsu Collaborative Innovation Center of Meat Production and Processing.

## Conflict of Interest

The authors declare that the research was conducted in the absence of any commercial or financial relationships that could be construed as a potential conflict of interest.
